# Transcription Factor Binding Sites Prediction Based on Modified Nucleosomes

**DOI:** 10.1371/journal.pone.0089226

**Published:** 2014-02-21

**Authors:** Mohammad Talebzadeh, Fatemeh Zare-Mirakabad

**Affiliations:** Department of Mathematics and Computer Science, AmirKabir University of Technology, Tehran, Iran; George Mason University, United States of America

## Abstract

In computational methods, position weight matrices (PWMs) are commonly applied for transcription factor binding site (TFBS) prediction. Although these matrices are more accurate than simple consensus sequences to predict actual binding sites, they usually produce a large number of false positive (FP) predictions and so are impoverished sources of information. Several studies have employed additional sources of information such as sequence conservation or the vicinity to transcription start sites to distinguish true binding regions from random ones. Recently, the spatial distribution of modified nucleosomes has been shown to be associated with different promoter architectures. These aligned patterns can facilitate DNA accessibility for transcription factors. We hypothesize that using data from these aligned and periodic patterns can improve the performance of binding region prediction. In this study, we propose two effective features, “modified nucleosomes neighboring” and “modified nucleosomes occupancy”, to decrease FP in binding site discovery. Based on these features, we designed a logistic regression classifier which estimates the probability of a region as a TFBS. Our model learned each feature based on Sp1 binding sites on Chromosome 1 and was tested on the other chromosomes in human CD4+T cells. In this work, we investigated 21 histone modifications and found that only 8 out of 21 marks are strongly correlated with transcription factor binding regions. To prove that these features are not specific to Sp1, we combined the logistic regression classifier with the PWM, and created a new model to search TFBSs on the genome. We tested the model using transcription factors MAZ, PU.1 and ELF1 and compared the results to those using only the PWM. The results show that our model can predict Transcription factor binding regions more successfully. The relative simplicity of the model and capability of integrating other features make it a superior method for TFBS prediction.

## Introduction

Gene regulation is affected by the binding of transcription factors (TFs) to regulatory sequences in DNA. Recognition of transcription factor binding sites (TFBSs) improves insights into the genes regulated by a TF. These target genes combined with their expression data can be used to elucidate transcriptional regulatory networks and transcription regulation mechanisms [1],[2]. Due to significant sequence variation in the binding sites of a TF, transcription factor binding site prediction is still known as a difficult and central problem in computational biology [1]–[10]. This problem uses motif prediction in which a set of annotated binding sites and a new sequence are given as input, with the goal of finding similar binding site on the sequence [5], [8].

Chromatin immunoprecipitation followed by high throughput sequencing (ChIP-Seq) [11]–[14] and array hybridization (ChIP-chip) [15] experiments, are two promising high throughput technologies for identification of TF binding locations [13], [15]–[21]. These technologies have been successfully used to map binding locations in several organisms but some properties of these experiments such as being tissue and condition specific, the availability of antibodies for TFs under study, and the expense of the experiments have made them useful only for a limited number of TFs [2], [7], [10]. Therefore, utilization of computational approaches to identify binding sites seems inevitable [1]–[5], [7], [9], [10].

Binding sites of a TF can be represented as a consensus sequence or a position weight matrix (PWM). Despite the ease of visual interpretation, variations in nucleotide composition of binding sites make consensus sequences an unsuitable approach to represent TFs [4], [6]. So, the most common methods apply PWMs for TFBS representation instead of consensus sequence [8].

TFs usually bind to short (4–12 bp) DNA sequences. The repetitive nature of DNA causes the binding sites to occur at many locations throughout the eukaryotic genome, of which only a small number are involved in the regulatory processes of the cell. These considerations make motif scanning i.e. searching DNA sequence for matches with a PWM, to be highly uncertain and to produce a high frequency of false positive predictions [3], [5], [9]. This problem is more evident in mammalian genome since cis-regulatory elements are usually kilo bases away from target genes, making it necessary to search large regions, which in turn leads to an increase in false positives [7]. These challenges undermine the use of motif scanning as a standalone method for TFBS prediction.

Common PWMs do not take into account higher order dependencies between nucleotides, thus it is believed that developing better models for binding sites and utilizing higher order background models will improve the performance of motif prediction[22]–[24]. Construction of such complex models has proved to be challenging [25], so the use of additional data sources in the context of TFBS prediction is attracting more attention [5].

Eukaryotic DNA is packaged into nucleosomes and forms local structures of chromatin [26], [27]. Dynamic changes in **chromatin structure** through **post*-*translational modifications of histones,** restrict accessibility of DNA for TFs [3], [28]. Several studies have shown that TFs binding to genomic regions are associated with various histone modification levels [20], [29]–[31]. According to these observations, several studies have developed frameworks to improve TFBSs prediction accuracy using a limited number of epigenetic experimental assays [1]–[3], [7], [9], [10]. They considered the numbers of different histone modification tags as additional information sources for improving the prediction accuracy.

Nozaki et al. [28] showed that nucleosomes harboring histone modifications like H3K4me1, H3K4me2, H3K4me3 as well as the histone variant H2A.z have an aligned and periodic pattern around broad promoters. They concluded that this might be due to the accessibility of TFs to DNA.

In this study, we were interested in using information from the positions and distribution of modified nucleosomes to improve the performance of TFBS prediction. We have examined the effects of two features “modified nucleosomes neighboring” (MNN) and “modified nucleosomes occupancy” (MNO) around TFBSs.

The MNN feature considers the closest distance from a TFBS to the nearest nucleosome harboring a specific histone modification. The MNO feature represents the total number of nucleosomes containing a histone modification around the binding sites of a transcription factor.

To investigate these features, we considered 21 histone methylation modifications [30]. For each feature (MNN, MNO) a set of values corresponding to different modifications were computed based on Sp1 binding sites on Chromosome 1 in human CD4+T cells. Then, these values were applied as a training set in a logistic regression classifier (LRC). The rest of (Chromosome 2–22) autosomes and two sex chromosomes were used as a test set to show that these features are capable of predicting Sp1 binding regions on other chromosomes. We found that only 8 out of 21 histone modifications, namely H2A.z; H3K4me1, H3K4me2, H3K4me3; H3K9me1; H3K27me1; H4K20me1 and H2BK5me1 are strongly correlated with transcription factor binding regions.

We next designed a second model to search a genome for TFBSs based on the features (MNN, MNO) combined with using the PWM. We applied this model on MAZ, PU.1 and ELF1 TFs and compared the results with the common model using PWM alone. The results show that false positives are significantly decreased with only a minor decrease in true positives.

## Results and Discussion

In this section, we introduce two features based on the modified nucleosomes which are effective for distinguishing transcription factor binding regions from random ones. We call these features “modified nucleosomes neighboring” (MNN) and “modified nucleosomes occupancy” (MNO).

At first, we analyzed the MNN feature using the general transcription factor Sp1 in human resting CD4+T cells. Through the evaluation of the MNN feature, eight significant histone modifications were identified for TFBS prediction. Next, the MNO feature was applied on these eight marks to show that the occupancy of modified nucleosomes is also predictive of true binding regions. Finally, a model was designed which integrates PWM with the MNN and MNO features to improve prediction of the MAZ, PU.1 and ELF1 binding sites.

### 1. Benefit of “Modified Nucleosomes Neighboring”

In this part, we would like to show that the vicinity of nucleosomes containing a certain histone modification is useful for predicting TFBSs. At first, we extracted Sp1 binding sites from Chromosome 1 in human CD4+T cells. Then, for each histone modification, the distance between the Sp1 binding sites and the nearest nucleosomes containing the modification was calculated and considered as a training set in the LRC model (see Methods). Next, 21 (Chromosome 2–22) autosomes and two sex chromosomes were binned into 1-kb non overlapping intervals and used as a test set for the model (see Methods). Intervals on these chromosomes were then predicted by the model as Sp1 binding regions [1].

**Figure 1:**
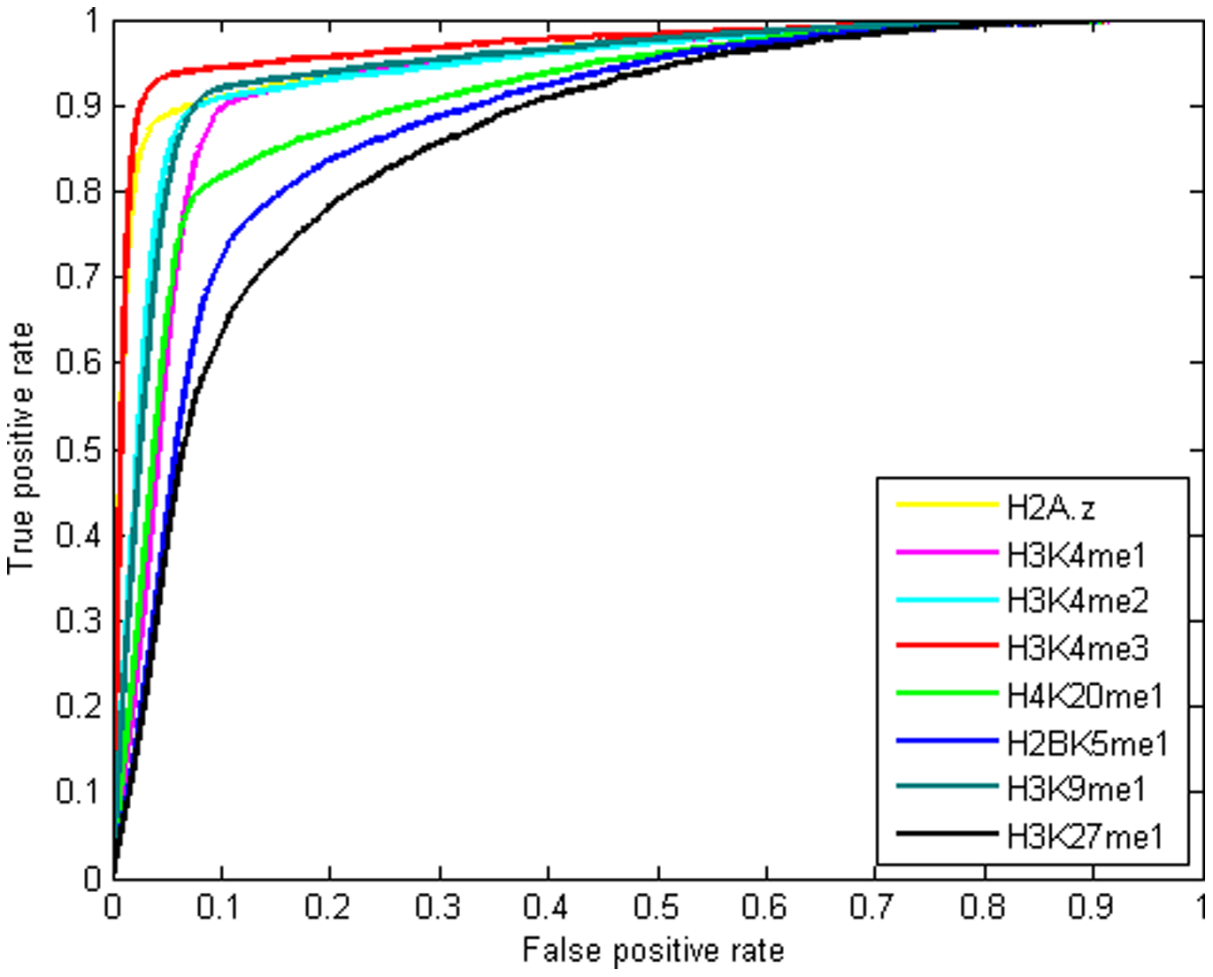
ROC curves for predicting the binding regions of Sp1using the MNN feature. ROC curves for 21 LRCs trained on individual histone modifications for prediction of Sp1 binding regions, using the MNN feature. The LRCs corresponding to each histone modification were trained on Chromosome 1 and tested on Chromosome 2 to 22 and two sex chromosomes. The LRCs assign a score to each interval. Predictions of binding regions are based on these scores. These curves show that the MNN feature is predictive of binding regions even when no PWM score is used. The x-axis is the false positive rate and the y-axis is the true positive rate. Shown are the curves of the most predictive modifications. ROC curves for the rest 13 modifications can be found in Figure S1.

**Figure 2:**
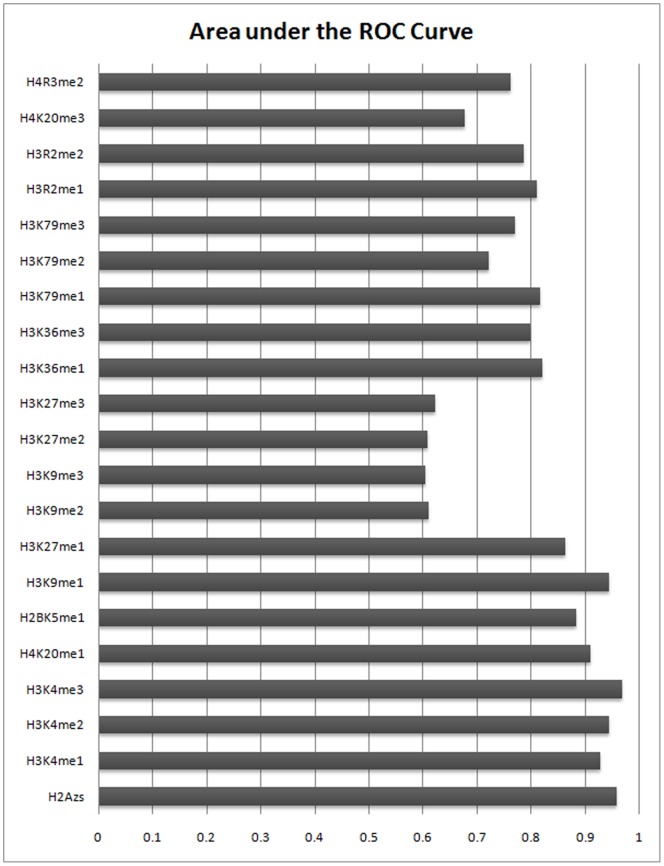
AUC values corresponding to different histone modifications for predicting the binding regions of Sp1 based on the MNN feature. Results are shown for predicting the binding sites of Sp1 in CD4+T cells using the MNN feature. The height of each bar corresponds to the Area under the ROC curves. Certain modifications are more predictive for true binding regions. Comparing the results with using the PWM alone (Figure3) clearly shows that the MNN feature, especially for certain modifications, can be used as an informative feature for TFBSs prediction.

This task was repeated for nucleosomes containing each of the 21 histone modifications. Figure 1 (and Figure S1 and Table S1) and Figure 2 show ROC curves and AUC values, respectively for 21 LRCs associated with different histone modifications. These figures show the results of evaluations averaged on the test set (21 autosomes and two sex chromosomes).

**Figure 3:**
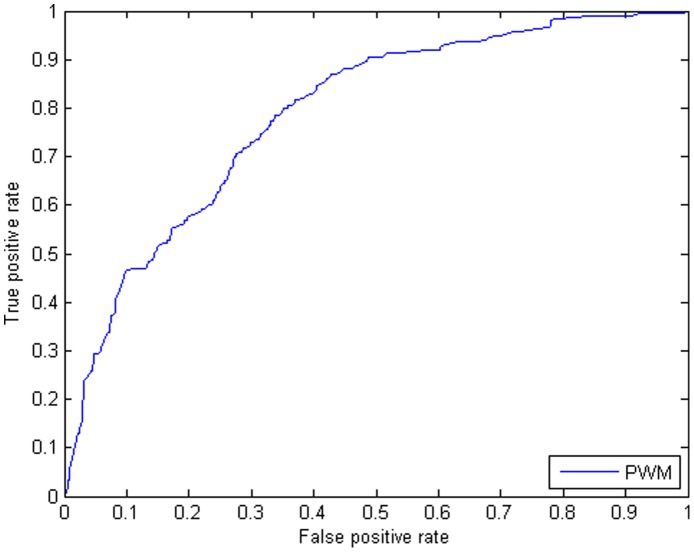
The standard ROC curves for the traditional motif scanning method with a zero order background model. Result is shown for predicting the test set (Chromosome 2 to Chromosome 22 and two sex chromosomes) binding regions of Sp1 in CD4+T cells using the PWM. AUC value corresponding to this curve is 0.7880.

To compare the accuracy of this feature with PWM, test set intervals were scored using an Sp1 PWM constructed based on binding sites on Chromosome 1 (see Methods). The ROC curve and AUC value of PWM scanning is shown in Figure 3.

Comparison of the PWM AUC value with previous results clearly shows that the MNN feature, especially for certain marks, greatly outperforms the traditional scanning approach. This demonstrates the usefulness of our proposed MNN feature in recognition of target sites.

These figures clearly show that using the MNN feature greatly reduces the number of false positives with a minor decrease in sensitivity. However histone modifications do not contribute equally in prediction improvements. Comparing AUC values show that seven types of histone modification, H3K4me1, H3K4me2, H3K4me3, H4K20me1, H2BK5me1, H3K9me1, H3K27m1 as well as histone variant H2A.z have a significant effect on improvement. We refer to these eight modifications as top marks.

It has been shown that 21 histone modifications can be classified into three groups [21]. Active modifications are related to active genes and enhancers. Repressive modifications are connected with repressed genes and heterochromatin and moderate modifications have no preference toward any of activated or repressed genes. Table 1 shows each modification and its assigned category.

**Table 1:** Histone modification types.

Histone modification	Modification type
H2A.Z	Active
H3K4me1	Active
H3K4me2	Active
H3K4me3	Active
H3K9me1	Active
H3K9me2	Repressive
H3K9me3	Repressive
H3K27me1	Active
H3K27me2	Moderate
H3K27me3	Repressive
H3K36me1	Moderate
H3K36me3	Active
H3K79me1	Moderate
H3K79me2	Moderate
H3K79me3	Moderate
H3R2me1	Moderate
H3R2me2	Moderate
H4K20me1	Active
H4K20me3	Moderate
H4R3me2	Moderate
H2BK5me1	Active

Each modification is clustered into active, repressive or moderate type based on their association with active or repressed genes. Moderate marks show a dual tendency toward active and repressed genes.

Eight predicted top marks using our model are among the active modifications. Modifications with weak prediction accuracies (H3K9me2, H3K9me3 and H3K27me3) can be seen in the repressive group. The other histone modifications, with average prediction accuracies, appear in the moderate modifications. Among these moderate marks, H3K27me2 and H4K20me3 have the lowest AUC values. In contrast, H3K79me1 and H3K36me1 show relatively acceptable accuracies with AUC≈0.79. From these observations, we conclude that H3K27me2, H4K20me3 as well as other modifications in the moderate group with low AUC values have a tendency toward repressed genes. On the other side, highly predictive modifications can be connected to the activation of transcription. These findings are in line with previous studies that H4K20me3 is associated with heterochromatin, H3K36me1 shows a slight tendency toward active genes and H3K27me2 signals are more prevalent at silent promoters [30], [32]–[34]. The signals and functional consequences of H3K79me1 are not well studied but our results predict a slightly active tendency for this mark.

Putting it all together, we reason out that active histone modifications are more predictive for TFBSs prediction with the exception of H3K36me3 mark. Studies have shown that this epigenetic mark is considered as an active mark only when this mark lies in the coding region of a gene, and a repressing mark in the promoter region [26], [30]. This particular distribution of H3K36me3 may explain a cause for poor predictive power of this modification.

The histone code hypothesis [35] suggests that a combination of histone modifications affects gene regulation. So, we analyzed whether combining **histone modifications can predict Sp1 binding sites better than single marks.**

We considered three combinations among histone modifications as follows: 1) combining all 21 modifications, 2) combining the eight top marks 3) integrating H3K4me3 and H2A.Z data. We chose H3K4me3 and H2A.z because they have the largest AUC values among top 8 marks (Figure 1).

**Figure 4:**
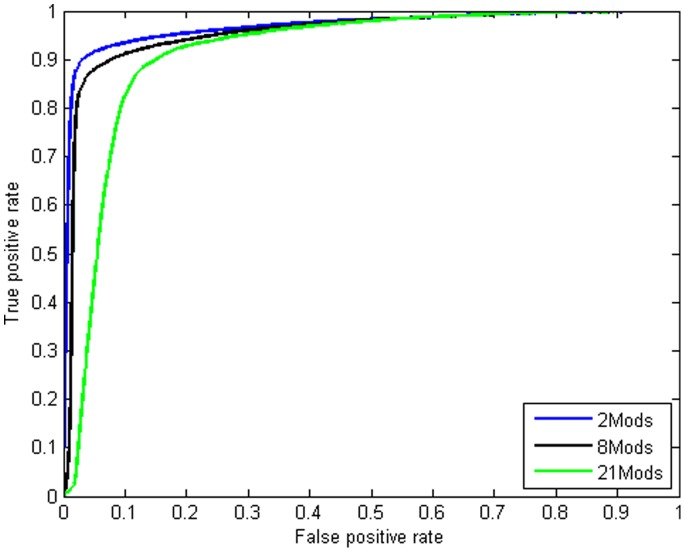
Improvement in Sp1 binding site prediction by combining MNN data from different modifications. ROC curves for a number of different methods for predicting bound locations. Results of predictions made by combining all 21 modifications (green line); 8 modifications (black line) and integrating H2A.Z and H3K4me3 data (blue line). Comparing this figure with Figure 1 shows that applying the LRCs to the data of single modifications perform better than those LRCs trained with the combination of histone modifications. This may be due to the fact that the predictive ability for distinguishing true target regions is redundantly encoded among histone marks.

In comparison with using single modifications, integrating more marks is expected to enhance the accuracy of predictions. The results show that the most predictive single histone modification for Sp1 is H3K4me3 with an AUC value of 0.9683. The AUC values for 21, 8 and 2 combined histone modifications are 0.9149, 0.9545 and 0.9682, respectively (Figure 4).

The failure of combining modifications may be due to the fact that histone modifications are closely correlated and there is a data redundancy among them [36].2.

### 2. Benefit of “Modified Nucleosomes Occupancy”

In this part, we are interested to show that the combination of the top eight marks can be used as an acceptable approach in TFBSs prediction. So, we considered another feature called modified nucleosomes occupancy which represents the total number of nucleosomes harboring a histone mark in the binding regions. Therefore, the total number of nucleosomes containing the top 8 marks in 1-kbp regions flanking Sp1 sites on Chromosome1 were computed and applied as a training set in the LRC (see Methods). As before, Sp1 bound regions on 21 autosomes and two sex chromosomes were employed to evaluate the predictive power of the feature. Figure S2 shows that utilizing occupancies of nucleosomes can be informative in actual binding sites prediction.

**Figure 5:**
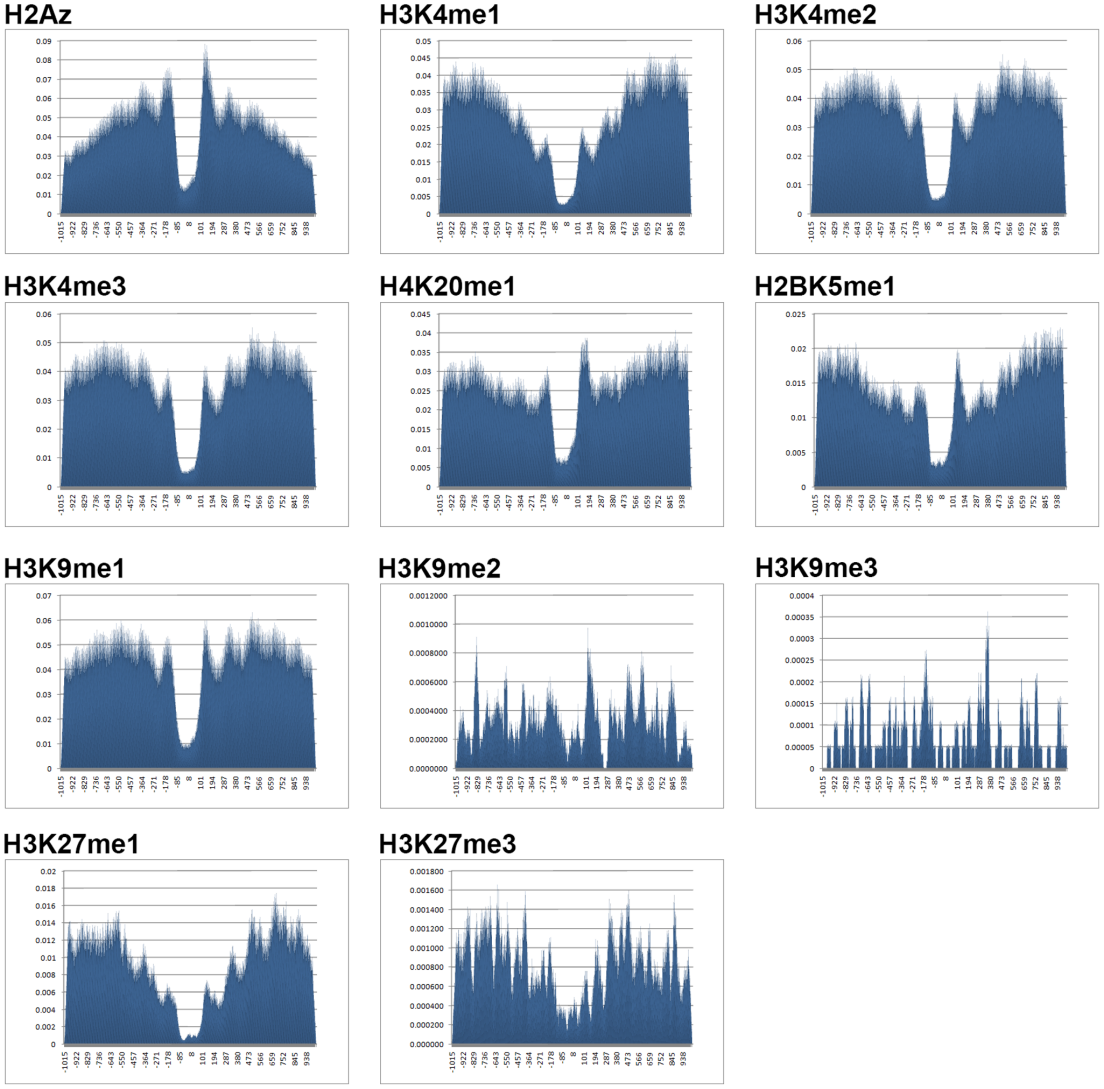
Distributions of nucleosome positions around Sp1 binding sites. Distributions of the central positions of nucleosomes for the top 8 marks and 3 repressive marks around Sp1 binding sites on the genome. The x-axis shows genomic positions with respect to central position of Sp1 binding sites (from −1015bp to +1015bp). The positions of nucleosomes are defined as the positions from −15 bp to 15 bp with respect to the center of the nucleosome. Active marks are highly enriched around binding sites and show a bimodal distribution around these sites. A nucleosome free region with respect to central position of binding sites is also observable in all top marks.

To provide evidence why certain modifications are more predictive than the others, the ratio of nucleosome containing top 8 marks and three repressive histone modifications (as a control) were calculated at each position around binding sites of Sp1 (see Methods). We found that nucleosomes containing the top 8 marks are enriched and show a bimodal pattern around Sp1 binding sites. A nucleosome depleted region with respect to the center of the binding sites is evident in active marks (Figure 5). This bimodal distribution may indicate TFs compete with nucleosomes to access DNA. We can consider these nucleosome free regions flanked by modified nucleosomes as landing sites which direct TFs into the true binding locations.

### 3. Evaluation of the Suggested Features on the other Transcription Factors

Studies have suggested that epigenetic data show a general binding tendency for TFs in genomic regions and thus are not specific to a given TF [3]. Therefore, the assigned scores to the test set chromosomes based on Sp1 MNN and MNO features, may be predictive of the other TFs as well.

To investigate this hypothesis, we developed a new model to predict the binding regions of three additional transcription factors MAZ, ELF1 and PU.1 based on MNN and MNO features.

**Figure 6:**
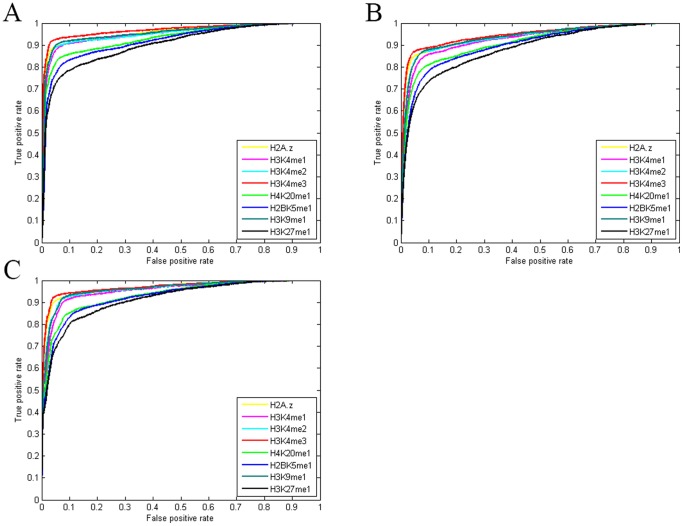
ROC curves for predicting the binding locations of MAZ, ELF1 and PU.1 using the MNN feature combined with the PWM scores. Results are shown for predicting the binding locations of A) MAZ, B) PU.1, C) ELF1 using the MNN feature with different histone modifications, combined with the PWM scores. The final score assigned to each region is 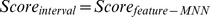 as introduced in the Methods. ROC curves for the rest of the 13 modifications can be found in Figure S3. Comparing this figure with Figure S4, S5, S6 clearly demonstrate the usefulness of the MNN feature for prediction of binding locations.

First, each test set interval was scored based on the MNN feature, trained on Sp1 binding sites. Then, these scores were combined with the PWM of the corresponding TF (see Methods). These scores were used to predict binding regions related to each TF. ROC Curves for these three TFs and 21 modifications are shown in Figure 6 (and Figures S3). As expected, Comparing these figures with ROC curves of PWM scanning (Figures S4, S5, S6) confirms the higher predictive power of the top eight identified modifications. The AUC values are illustrated in Table S2.

We further evaluated the effect of the MNO feature on prediction of these TFs (Figure S7 and Table S3). Like the MNN feature, the nucleosome occupancies combined with PWM scores, significantly enhance predictions over using PWM alone. This confirms the usefulness of epigenetic data in the context of TFBS prediction.

Finally, we illustrated the ratio of marked nucleosome at each position around binding sites of MAZ, PU.1 and ELF1 (Figures S8, S9, S10) and observed the bimodal patterns of the top 8 modified nucleosomes distributed around the central position of binding sites in these three TFs as well.

## Conclusions

By using a probabilistic approach, we discovered that using the genomic position of modified nucleosomes can be informative for predicting the binding locations of TFs. We first showed that the vicinity of modified nucleosomes around TF binding sites combined with PWM can enhance the performance of predictions over using PWM alone. Then, we observed that eight types of histone modifications correlate more highly with TFBSs. Using these eight modifications, we investigated the nucleosomes occupancy around the binding sites, and again showed that this feature is also correlated to the target regions of TFs. The analysis of the modified nucleosomes distribution around binding sites revealed that these nucleosomes show a bimodal distribution with a depleted region right on the center of binding sites.

In this study, we used two features, namely “Modified Nucleosome Neighboring” and “Modified Nucleosome Occupancy” to analyze whether the spatial distribution of nucleosomes are informative for TFBS prediction. The proposed features as well as the classifier can be easily applied to other TFs to evaluate how well these features will perform in prediction processes.

Here, we only analyzed the role of 21 histone methylation in TFBS prediction. As more and more genome-scale histone modification data sets become available, more complex features related to the distribution of nucleosomes may be defined and used to uncover the actual patterns that the modified nucleosomes take around binding sites.

We believe that our study is a step toward understanding epigenetic regulation of target genes of TFs and inferring how epigenetic modifications influence and recruit regulatory proteins.

## Materials and Methods

### 1. Transcription Factor Binding Sites

Genome wide position of Sp1, MAZ, PU.1 and ELF1 binding sites were obtained from FANTOM 4 (http://fantom.gsc.riken.jp/4/download/GenomeBrowser/hg18/TFBS_CAGE/allsites_cage_tfbs_feb09_latest.gff.gz) [37].

### 2. Nucleosome Position Detection and Dataset

The genomic position of the 21 modified nucleosomes in human resting CD4+T cells were obtained from [30]. These data show the genomic position of unambiguously mapped sequence tags from chromatin immunoprecipitation followed by high-throughput sequencing (ChIP-Seq) experiments. We used the NPS package [21] to determine the genomic positions of nucleosomes corresponding to the short sequence tags. The March 2006 human genome (NCBI Build 36.1, hg18 assembly) was used as a reference genome.

### 3. Distribution of Nucleosome Positions around Transcription Factor Binding Sites

The distributions of the nucleosomes from −1 kb to +1 kb with respect to the center of TF binding sites from the positions identified in the previous steps were calculated by dividing the number of nucleosomes at each position by the number of binding sites. Genomic positions from −15 bp to 15 bp with respect to the central positions of the nucleosomes are assumed as the genomic positions where the nucleosomes exist. The distributions of nucleosomes near Sp1, MAZ, PU.1 and ELF1 binding sites were separately calculated [28].

### 4. Position Weight Matrix Representation

A zero order background model represented by [38] was used to construct the matrix based on Sp1 binding sites extracted from Chromosome1. The number of nucleotides in each position was calculated and converted to a frequency as follows:
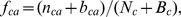
where 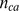 and 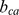 are the real counts and pseudocounts of nucleotide 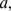 respectively in position 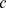. The total number of real counts and pseudocounts are called 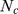 and 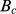 in the position, respectively. Here we consider 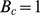 and 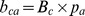 where 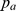 is the background frequency of base 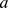. These frequencies are then converted to a log odd score as follows:
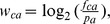
where 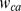 is the matrix value of base 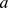 in position 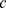. The scores given to a binding site are converted to a relative unit scale by:
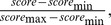
where 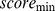 and 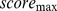 are the sums of minimum and maximum scores in each column of constructed PWM, respectively. This strategy was used to construct MAZ, PU.1 and ELF1 PWMs.

### 5. Scanning and Scoring Regions by PWM

All chromosomes except Chromosome 1, which is used as a training set, were binned into non-overlapping 1 kb intervals. Every base 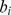 in each interval was scored by PWM. So, two scores 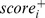 and 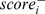 were assigned to the base 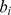 corresponding to two sub sequences on positive and negative strands. The score of each interval is finally represented by:
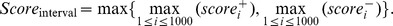



According to the constructed PWM, a score is assigned to each interval for each TF under study.

### 6. Training Set

For the modified nucleosome neighboring feature (MNN), the distances from the center of Sp1 binding sites on Chromosome 1 to the central positions of the nearest nucleosomes containing a specific modification were computed. These single values were used to train 21 LRCs corresponding to 21 histone modifications. Combinations of these values were used to train the LRCs corresponding to 21, 8 and 2 combined modifications.

For the modified nucleosome occupancy feature (MNO), another LRC was constructed, in which the number of modified nucleosomes of certain types (the top 8 marks) were computed in1-kbp regions flanking the center of Sp1 binding sites. These 8 obtained values were used as features to train the LRC.

For both MNN and MNO features, corresponding to each positive location in the training set, two random positions in non-gapped regions of the Chromosome 1 were selected as negative control. For these positions the MNN and MNO features corresponding to different histone modifications were computed the same as above.

### 7. Test Set

Each interval from 21 autosomes and two sex chromosomes containing the center of a reported binding site was considered as a true binding location and the others as false binding regions. For each region, values corresponding to the MNN and MNO features were computed as follows. For the modified nucleosome neighboring feature (MNN), the closest distance from the center of the region to the nearest nucleosome containing a specific modification was computed. This task was done for each interval and for each 21 different modifications. Thus, for each interval 21 values corresponding to 21 different histone modifications were obtained. Finally each single value or combinations of these values, as described in the results, were inserted into the LRC model as a test set. The LRC classified the intervals as binding or non-binding locations.

For the nucleosome occupancy feature (MNO), only top 8 marks (recognized through evaluation of the MNN feature) were considered and in each test set interval, the total numbers of nucleosomes containing each of these top modifications were calculated separately. So, 8 values were assigned to each interval. These 8 values were inserted into the LRC for evaluation purposes. The LRCs assigned a score to each interval which showed the Sp1 binding probability to that interval.

### 8. Evaluating the Features on MAZ, PU.1 and ELF1

Since Sp1 is a well-known and ubiquitous protein and has been reported to bind practically everywhere in the human genome [5], the same LRCs trained on Sp1 were used to score test set intervals. Then, these scores were made specific to each MAZ, PU.1 and ELF by integrating these values with the PWMs corresponding to each factor (see below).

### 9. Integrating Logistic Regression with Position Weight Matrix

For each test interval the score is computed as follows:
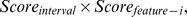
where 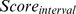 and 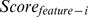 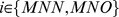, are calculated based on the PWM of the TF under study and the LRC model trained on SP1.

### 10. Logistic Regression Classifier

We used a logistic regression classifier (LRC) to integrate multiple data sources. This classifier maps a single or a set of computed features to a score which represents the probability of a TFBS.

A TFBS prediction can be represented as a binary classification problem.The LRC hypothesis function used for prediction is defined as 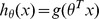 where 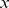 is the vector of input features. The vector of parameters 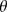 (also called weights) can be estimated based on the training examples. In this study we chose the logistic function, 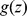, to be a sigmoid function:
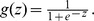



So, 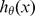 is always a real number between 0 and 1(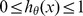). Here 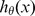 shows the probability of a region being a binding site:
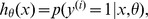
where 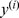 shows the 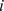 th region which can be the target of a TF. To estimate the parameters 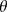
_,_ we define the cost function as:
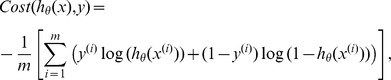



Where 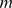 is the number of training examples and 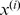 is the vector of input features (the MNN or the MNO) computed for the 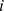 th interval. We call this function 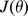. To Estimate the parameters 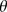, we need to minimize this function. The Matlab function 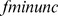 was used to minimize and estimate these weights. All calculations were done in Matlab R2012a. Preparation of the data was done in C# 2010.

## Supporting Information

Figure S1 **ROC curves for predicting the binding regions of Sp1 based on the MNN feature.** ROC curves are shown for 13 modifications with less predictive power for prediction of Sp1 binding regions on the test set. The MNN feature is used to train corresponding LRCs on Chromosome 1. Only scores assigned by the LRCs (without using PWM scores) are used to predict binding regions. The x-axis is the false positive rate and the y-axis is the true positive rate.
(TIF)

Figure S2 **ROC Curve of the modified nucleosome occupancy feature for prediction of the Sp1 target regions.** The ability of the LRC trained on the MNO feature-to differentiate between reported bound locations of Sp1 and random sites (AUC = 0.9413). Not only is the vicinity to modified nucleosomes but also the total number of these nucleosomes an appropriate identifier of true binding regions. The MNO feature is an eight dimensional vector (corresponding to top 8 marks), each element of which is the total number of nucleosomes containing a certain marks.
(TIF)

Figure S3 **ROC curves for predicting the binding locations of MAZ, ELF1 and PU.1 using the MNN feature combined with the PWM scores.** ROC curves are shown for the 13 modifications with less predictive power in A) MAZ, B) PU.1, C) ELF1. Each interval final score is a combination of MNN scores and PWM score corresponding to a TF under study. The ability of the LRCs, trained on Sp1 data, in predicting true binding regions of other TFs show that epigenetic modifications of nucleosomes are not specific to a certain TF and these modifications represent the general binding tendency of other TFs as well.
(TIF)

Figure S4 **The standard ROC curves for the traditional motif scanning method with a zero order background model.** Result is shown for predicting the binding regions of MAZ in CD4+T cells using the PWM. The AUC value corresponding to this curve is 0.7818**.**
(TIF)

Figure S5 **The standard ROC curves for the traditional motif scanning method with a zero order background model.** Result is shown for predicting the binding regions of PU.1 in CD4+T cells using the PWM. The AUC value corresponding to this curve is 0.7195**.**
(TIF)

Figure S6 **The standard ROC curves for the traditional motif scanning method with a zero order background model.** Result is shown for predicting the binding regions of ELF1 in CD4+T cells using the PWM. The AUC value corresponding to this curve is 0.7378**.**
(TIF)

Figure S7 **ROC Curve of modified nucleosome occupancy feature combined with the PWM Scores, corresponding to MAZ, ELF1 and PU.1.** Curves show the ability of the MNO feature incorporated with PWM scores to differentiate between reported bound locations of MAZ (Blue line), PU.1 (green line) and ELF1 (red line) and random sites. This figure compared to Figure S4, S5, S6, demonstrates the predictive power of the MNO feature combined with the PWM scores.
(TIF)

Figure S8 Distributions of modified nucleosome positions around MAZ binding sites on the genome. Repressive sites are shown as negative controls. The x-axis shows genomic positions with respect to central position of MAZ binding sites (from −1015bp to +1015bp). The positions of nucleosomes are defined as the positions from −15 bp to 15 bp with respect to the center of the nucleosome. Active marks are highly enriched around binding sites and show a bimodal distribution around these sites. A nucleosome free region with respect to central position of binding sites is also observable in all top marks.
(TIF)

Figure S9 Distributions of modified nucleosome positions around PU.1 binding sites. Repressive sites are shown as negative controls. The x-axis shows genomic positions with respect to central position of PU.1 binding sites.
(TIF)

Figure S10 Distributions of modified nucleosome positions around ELF1 binding sites. Repressive sites are shown as negative controls. The x-axis shows genomic positions with respect to central position of ELF1 binding sites.
(TIF)

Table S1 **AUC values of different histone modifications.** AUC values for predicting Sp1 binding regions on 21 (Chromosome 2–22) autosomes and two sex chromosomes using modified nucleosome neighboring as the only feature for enhancing predictions. Among top 8 marks, H2A.z and H3K4me3 are the most predictive modifications.
(DOCX)

Table S2 **AUC values corresponding to the ROC curves for different histone modifications.** AUC values for predicting three separate TF binding regions on the test set (21 autosomes and two sex chromosomes) using modified nucleosome neighboring incorporated with PWM scores for enhancing predictions.
(DOCX)

Table S3 **AUC values of model incorporating MNO feature and PWM scores for prediction of bound regions of 3 TFs.** AUC values corresponding to prediction made by using occupancy of 8 top marks combined with PWM scores.
(DOCX)
