# Supplementary figures and images for: Transcription Factor Binding Sites Prediction Based on Modified Nucleosomes
Source: PLoS One. 2014 Feb 21;9(2):e89226. doi: 10.1371/journal.pone.0089226 (PMC3931712; doi:10.1371/journal.pone.0089226)

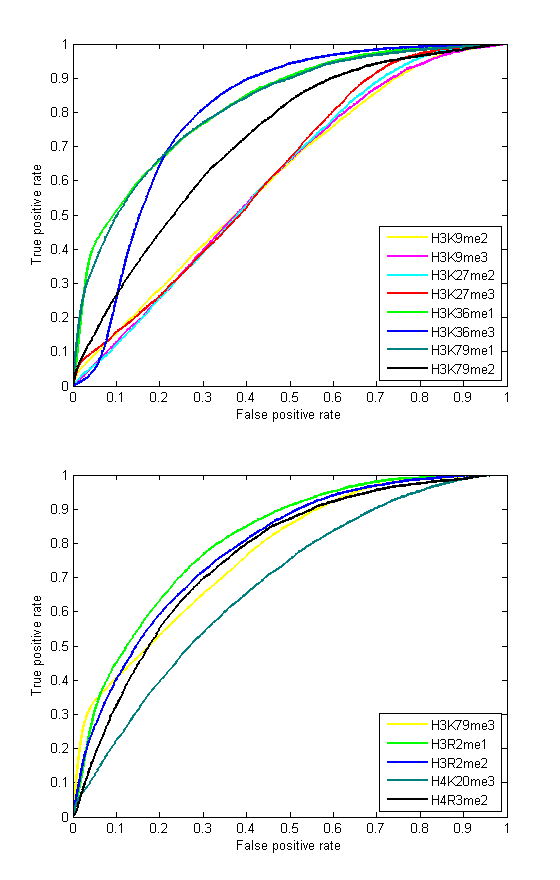

Supplement: Figure S1 — ROC curves for predicting the binding regions of Sp1 based on the MNN feature. ROC curves are shown for 13 modifications with less predictive power for prediction of Sp1 binding regions on the test set. The MNN feature is used to train corresponding LRCs on Chromosome 1. Only scores assigned by the LRCs (without using PWM scores) are used to predict binding regions. The x-axis is the false positive rate and the y-axis is the true positive rate. (TIF) [file pone.0089226.s001.tif]

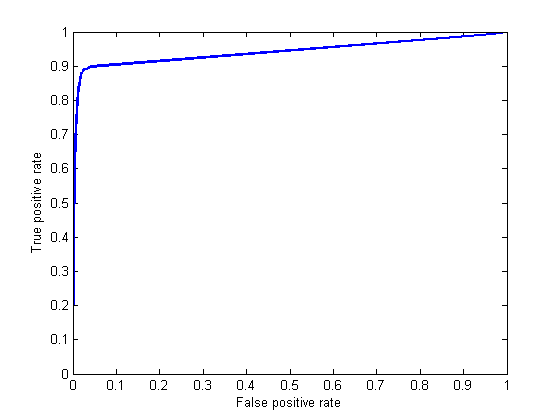

Supplement: Figure S2 — ROC Curve of the modified nucleosome occupancy feature for prediction of the Sp1 target regions. The ability of the LRC trained on the MNO feature-to differentiate between reported bound locations of Sp1 and random sites (AUC = 0.9413). Not only is the vicinity to modified nucleosomes but also the total number of these nucleosomes an appropriate identifier of true binding regions. The MNO feature is an eight dimensional vector (corresponding to top 8 marks), each element of which is the total number of nucleosomes containing a certain marks. (TIF) [file pone.0089226.s002.tif]

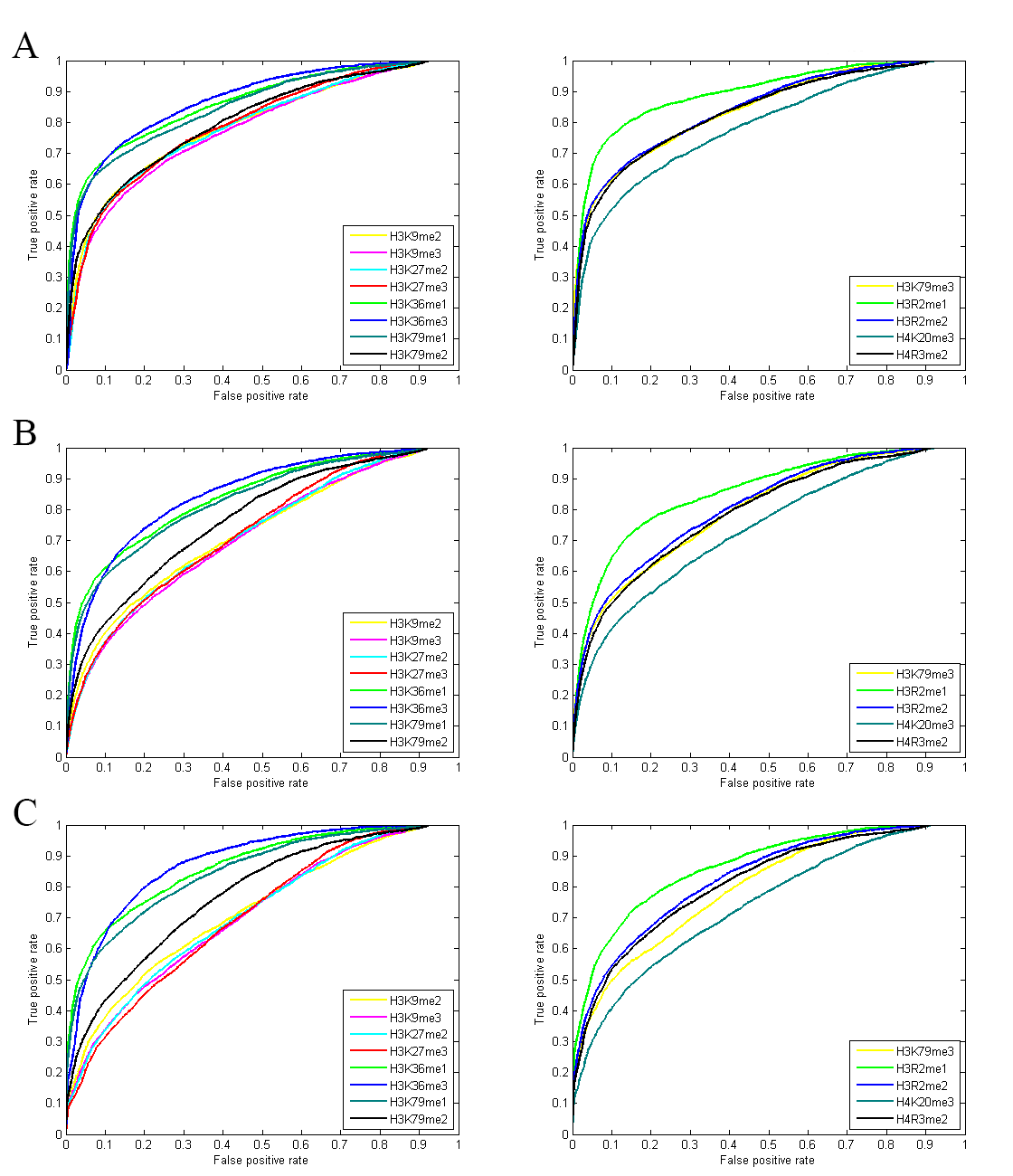

Supplement: Figure S3 — ROC curves for predicting the binding locations of MAZ, ELF1 and PU.1 using the MNN feature combined with the PWM scores. ROC curves are shown for the 13 modifications with less predictive power in A) MAZ, B) PU.1, C) ELF1. Each interval final score is a combination of MNN scores and PWM score corresponding to a TF under study. The ability of the LRCs, trained on Sp1 data, in predicting true binding regions of other TFs show that epigenetic modifications of nucleosomes are not specific to a certain TF and these modifications represent the general binding tendency of other TFs as well. (TIF) [file pone.0089226.s003.tif]

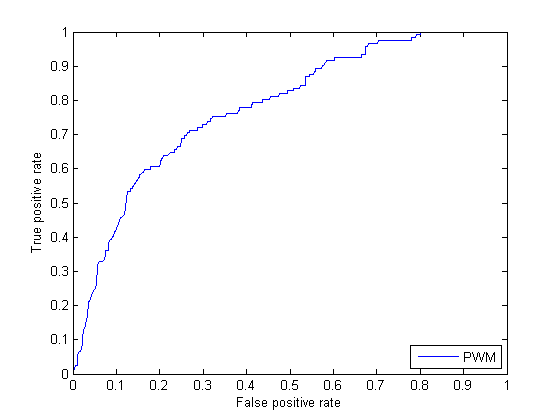

Supplement: Figure S4 — The standard ROC curves for the traditional motif scanning method with a zero order background model. Result is shown for predicting the binding regions of MAZ in CD4+T cells using the PWM. The AUC value corresponding to this curve is 0.7818. (TIF) [file pone.0089226.s004.tif]

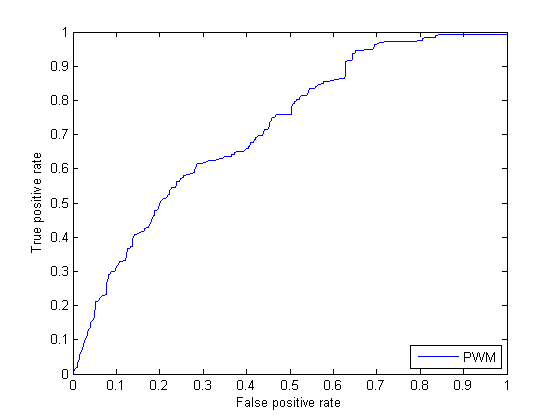

Supplement: Figure S5 — The standard ROC curves for the traditional motif scanning method with a zero order background model. Result is shown for predicting the binding regions of PU.1 in CD4+T cells using the PWM. The AUC value corresponding to this curve is 0.7195. (TIF) [file pone.0089226.s005.tif]

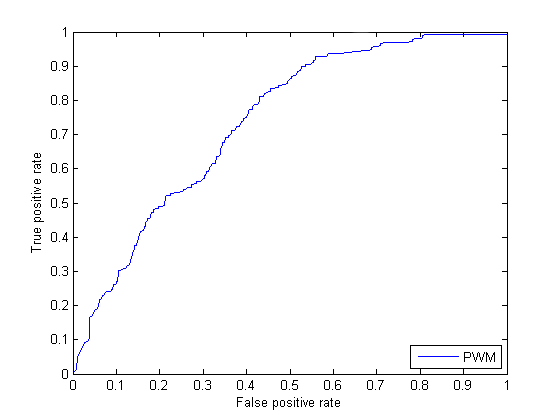

Supplement: Figure S6 — The standard ROC curves for the traditional motif scanning method with a zero order background model. Result is shown for predicting the binding regions of ELF1 in CD4+T cells using the PWM. The AUC value corresponding to this curve is 0.7378. (TIF) [file pone.0089226.s006.tif]

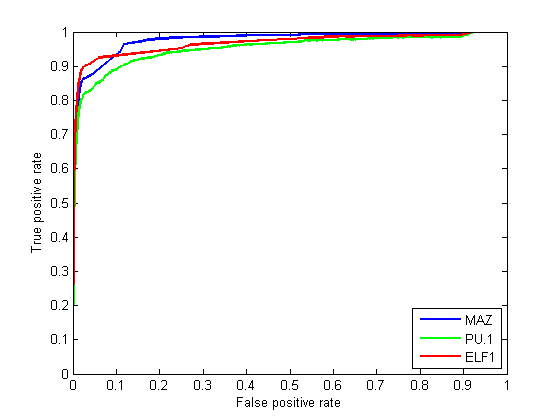

Supplement: Figure S7 — ROC Curve of modified nucleosome occupancy feature combined with the PWM Scores, corresponding to MAZ, ELF1 and PU.1. Curves show the ability of the MNO feature incorporated with PWM scores to differentiate between reported bound locations of MAZ (Blue line), PU.1 (green line) and ELF1 (red line) and random sites. This figure compared to Figure S4, S5, S6, demonstrates the predictive power of the MNO feature combined with the PWM scores. (TIF) [file pone.0089226.s007.tif]

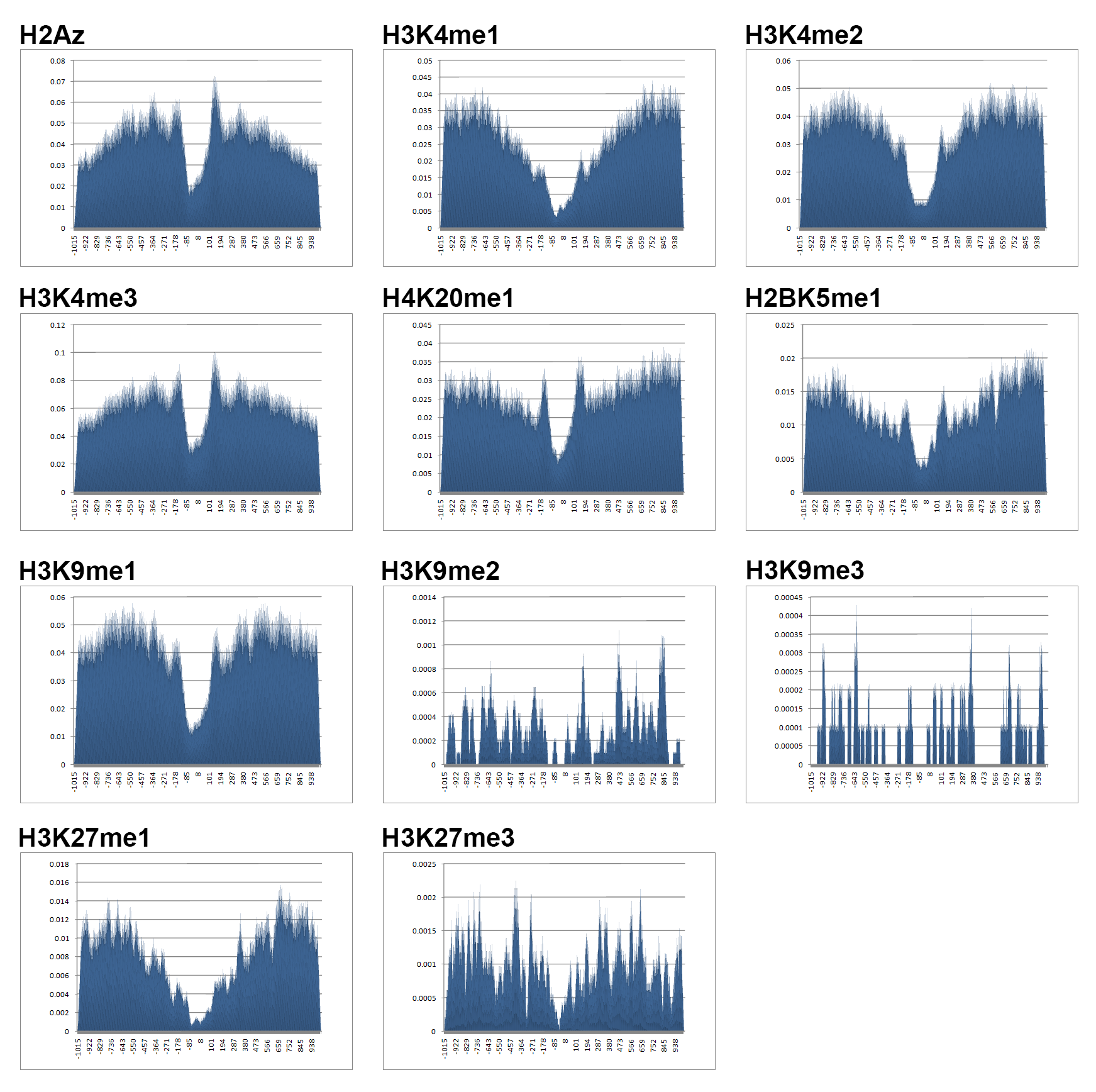

Supplement: Figure S8 — Distributions of modified nucleosome positions around MAZ binding sites on the genome. Repressive sites are shown as negative controls. The x-axis shows genomic positions with respect to central position of MAZ binding sites (from −1015bp to +1015bp). The positions of nucleosomes are defined as the positions from −15 bp to 15 bp with respect to the center of the nucleosome. Active marks are highly enriched around binding sites and show a bimodal distribution around these sites. A nucleosome free region with respect to central position of binding sites is also observable in all top marks. (TIF) [file pone.0089226.s008.tif]

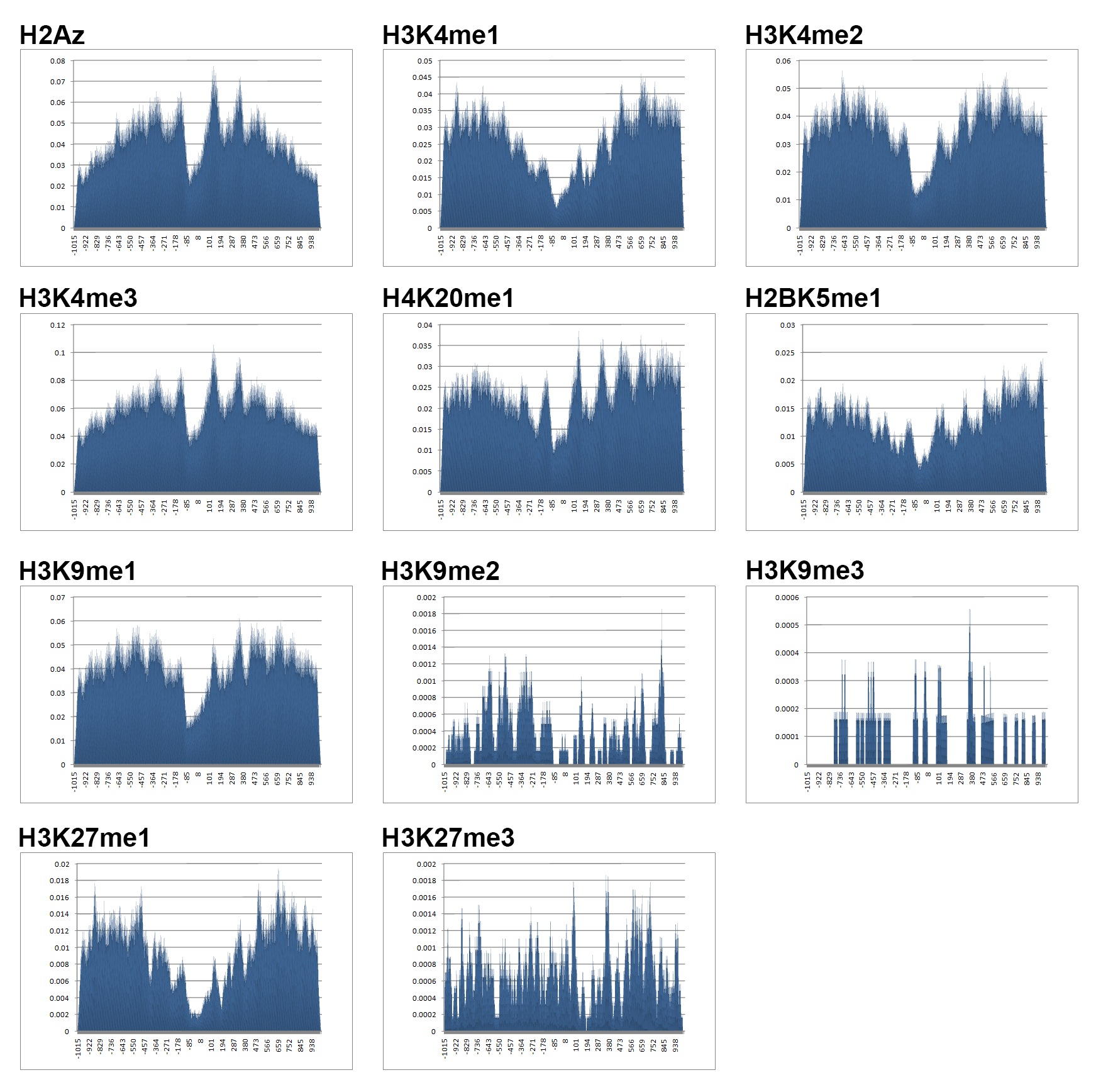

Supplement: Figure S9 — Distributions of modified nucleosome positions around PU.1 binding sites. Repressive sites are shown as negative controls. The x-axis shows genomic positions with respect to central position of PU.1 binding sites. (TIF) [file pone.0089226.s009.tif]

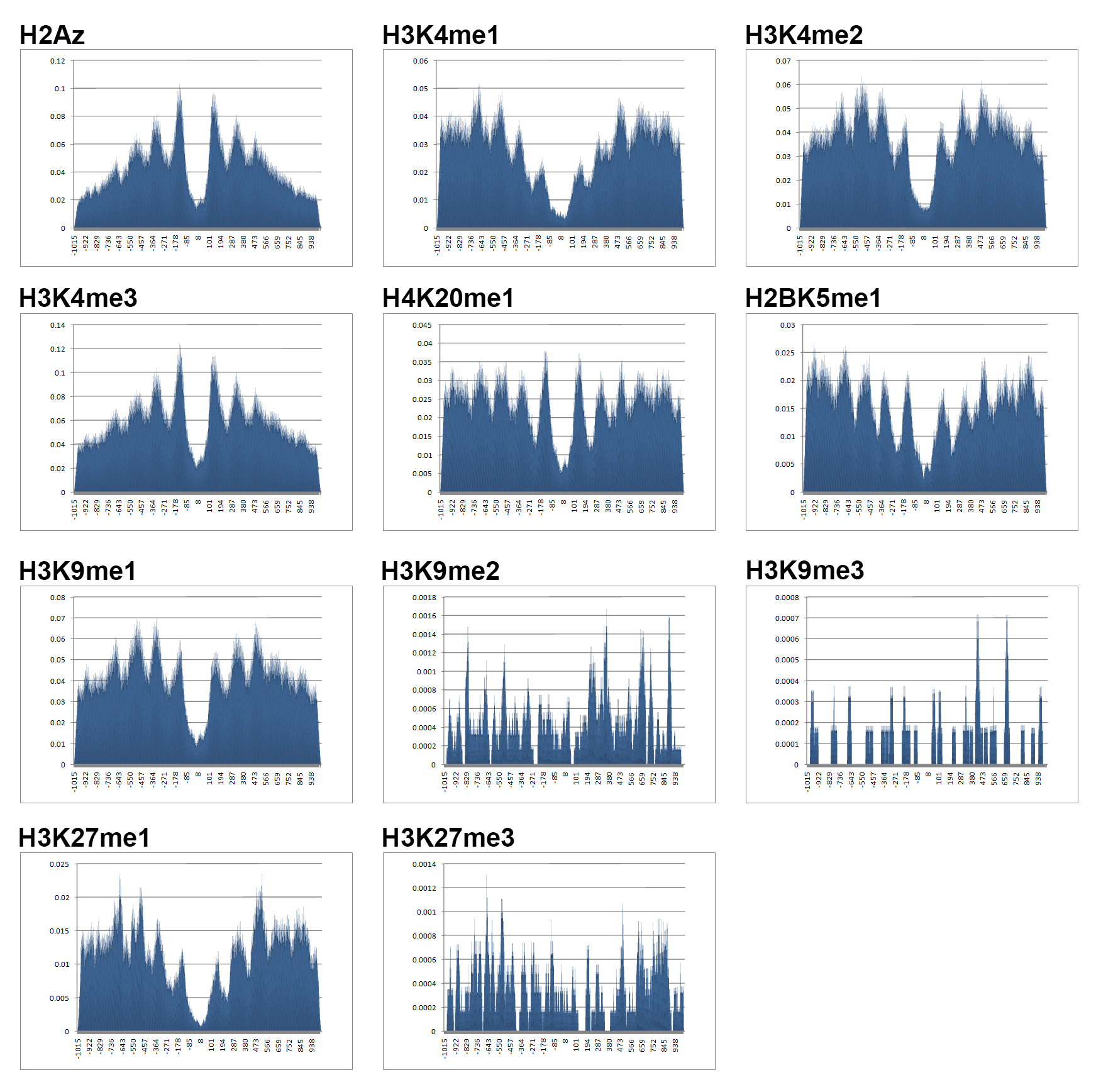

Supplement: Figure S10 — Distributions of modified nucleosome positions around ELF1 binding sites. Repressive sites are shown as negative controls. The x-axis shows genomic positions with respect to central position of ELF1 binding sites. (TIF) [file pone.0089226.s010.tif]
