# Supplementary material for: Transcription Factor Binding Sites Prediction Based on Modified Nucleosomes
Source: PLoS One. 2014 Feb 21;9(2):e89226. doi: 10.1371/journal.pone.0089226 (PMC3931712; doi:10.1371/journal.pone.0089226)
Supplement: Table S1 — AUC values of different histone modifications. AUC values for predicting Sp1 binding regions on 21 (Chromosome 2–22) autosomes and two sex chromosomes using modified nucleosome neighboring as the only feature for enhancing predictions. Among top 8 marks, H2A.z and H3K4me3 are the most predictive modifications. (DOCX) [file pone.0089226.s011.docx]

**Supplementary Table S1. AUC values of different histone modifications**

| Histone modification | AUC Value |
| --- | --- |
| H2A.z | **0.9571** |
| H3K4me1 | 0.9282 |
| H3K4me2 | 0.9429 |
| H3K4me3 | **0.9683** |
| H4K20me1 | 0.9097 |
| H2BK5me1 | 0.8829 |
| H3K9me1 | 0.9442 |
| H3K27me1 | 0.8625 |
| H3K9me2 | 0.6098 |
| H3K9me3 | 0.6048 |
| H3K27me2 | 0.6083 |
| H3K27me3 | 0.6213 |
| H3K36me1 | 0.8211 |
| H3K36me3 | 0.798 |
| H3K79me1 | 0.8164 |
| H3K79me2 | 0.7207 |
| H3K79me3 | 0.7693 |
| H3R2me1 | 0.8096 |
| H3R2me2 | 0.7867 |
| H4K20me3 | 0.6772 |
| H4R3me2 | 0.7614 |

AUC values for predicting Sp1 binding regions on 21 (Chromosome 2-22) autosomes and two sex chromosomes using modified nucleosome neighboring as the only feature for enhancing predictions. Among top 8 marks, H2A.z and H3K4me3 are the most predictive modifications.
