# Supplementary material for: Transcription Factor Binding Sites Prediction Based on Modified Nucleosomes
Source: PLoS One. 2014 Feb 21;9(2):e89226. doi: 10.1371/journal.pone.0089226 (PMC3931712; doi:10.1371/journal.pone.0089226)
Supplement: Table S2 — AUC values corresponding to the ROC curves for different histone modifications. AUC values for predicting three separate TF binding regions on the test set (21 autosomes and two sex chromosomes) using modified nucleosome neighboring incorporated with PWM scores for enhancing predictions. (DOCX) [file pone.0089226.s012.docx]

**Supplementary Table S2. AUC values corresponding to the ROC curves for different histone modifications.**

| Histone modification | AUC Values | | |
| --- | --- | --- | --- |
|  | MAZ | PU.1 | ELF1 |
| H2A.z | 0.9572 | 0.9402 | 0.9652 |
| H3K4me1 | 0.9482 | 0.9266 | 0.9524 |
| H3K4me2 | 0.9508 | 0.9349 | 0.9598 |
| H3K4me3 | 0.9671 | 0.9457 | 0.9689 |
| H4K20me1 | 0.9299 | 0.9096 | 0.9318 |
| H2BK5me1 | 0.9179 | 0.8989 | 0.9254 |
| H3K9me1 | 0.9545 | 0.9362 | 0.9601 |
| H3K27me1 | 0.9022 | 0.8828 | 0.9148 |
| H3K9me2 | 0.7894 | 0.7231 | 0.7198 |
| H3K9me3 | 0.7772 | 0.7112 | 0.7071 |
| H3K27me2 | 0.7872 | 0.7179 | 0.7105 |
| H3K27me3 | 0.7937 | 0.7254 | 0.7052 |
| H3K36me1 | 0.8646 | 0.8402 | 0.8656 |
| H3K36me3 | 0.8715 | 0.8478 | 0.8780 |
| H3K79me1 | 0.8534 | 0.8279 | 0.8478 |
| H3K79me2 | 0.8029 | 0.7669 | 0.7742 |
| H3K79me3 | 0.8391 | 0.7972 | 0.7951 |
| H3R2me1 | 0.8933 | 0.8556 | 0.8656 |
| H3R2me2 | 0.8421 | 0.8079 | 0.8273 |
| H4K20me3 | 0.7841 | 0.7338 | 0.7390 |
| H4R3me2 | 0.8332 | 0.7910 | 0.8120 |

AUC values for predicting three separate TF binding regions on the test set (21 autosomes and two sex chromosomes) using modified nucleosome neighboring incorporated with PWM scores for enhancing predictions.
