# Supplementary material for: Transcription Factor Binding Sites Prediction Based on Modified Nucleosomes
Source: PLoS One. 2014 Feb 21;9(2):e89226. doi: 10.1371/journal.pone.0089226 (PMC3931712; doi:10.1371/journal.pone.0089226)
Supplement: Table S3 — AUC values of model incorporating MNO feature and PWM scores for prediction of bound regions of 3 TFs. AUC values corresponding to prediction made by using occupancy of 8 top marks combined with PWM scores. (DOCX) [file pone.0089226.s013.docx]

**Supplementary Table S3. AUC values of model incorporating MNO feature and PWM scores for prediction of bound regions of 3 TFs.**

| Transcription factor | AUC Values |
| --- | --- |
| MAZ | 0.9750 |
| PU.1 | 0.9509 |
| ELF1 | 0.9667 |

AUC values corresponding to prediction made by using occupancy of 8 top marks combined with PWM scores.
